# Prevalence of Geographic Atrophy in Advanced Age-Related Macular Degeneration (AMD) in Daily Practice

**DOI:** 10.3390/jcm12144862

**Published:** 2023-07-24

**Authors:** Alaa Din Abdin, Machteld Devenijn, Roxana Fulga, Achim Langenbucher, Berthold Seitz, Hakan Kaymak

**Affiliations:** 1 Department of Ophthalmology, Saarland University Medical Center UKS, 66421 Homburg, Saar, Germany; 2 Internationale Innovative Ophthalmochirurgie, Breyer Kaymak Klabe Augenchirurgie, 40549 Düsseldorf, Nordrhein-Westfalen, Germany; 3 Institute of Experimental Ophthalmology, Saarland University, 66421 Homburg, Saar, Germany

**Keywords:** age-related macular degeneration, geographic atrophy, foveal involvement, intravitreal pegcetacoplan

## Abstract

Purpose: To investigate the prevalence of geographic atrophy (GA) in advanced age-related macular degeneration (AMD) and the proportion of eyes that would meet the indication criteria for treatment with the first intravitreal anti-C3 agent (pegcetacoplan). Methods: This retrospective cross-sectional study included all AMD patients who visited the Macular-Retina-Centre Oberkassel in 2021. Eyes were classified according to AMD stages. Eyes with GA were divided into two groups regarding foveal involvement. Baseline factors were compared between eyes with foveal GA (FGA) and eyes with non-foveal GA (NFGA) to identify predictive factors for foveal involvement. Results: A total of 2033 eyes from 1027 patients were included. AMD stage was early in 296 (14.5%) cases, intermediate in 368 (18.1%) cases, and advanced in 1249 (61.4%) cases. A total of 1204 (60%) eyes had GA [932 (77%) FGA and 272 (23%) NFGA], while 125 eyes (27.4% from eyes with advanced dry AMD) met the indication criteria for treatment with intravitreal pegcetacoplan. The proportion of eyes with neovascular AMD was significantly higher in the FGA group compared to the NFGA group [598 (64.2%) vs. 152 (55.8%), *p* = 0.01]. Conclusions: At least a quarter of eyes with advanced AMD would be suitable for the upcoming intravitreal pegcetacoplan therapy. Foveal involvement of GA in advanced AMD seems to be more likely in neovascular AMD than in dry AMD.

## 1. Introduction

Age-related macular degeneration (AMD) is a degenerative macular disease that affects 30 to 50 million people worldwide. It is recognised as the most common cause of legal blindness and visual impairment in Germany [1,2,3].

The prevalence of AMD is estimated to increase by about 50% to 288 million people between 2020 and 2040 [4]. This increase in the incidence of AMD has created a growing need for new AMD therapies [5].

The clinical manifestations of AMD range from drusen to geographic atrophy and/or vascular neovascularisation associated with exudation and haemorrhage. AMD has been classified based on examination findings including drusen, retinal pigment epithelium (RPE) abnormalities, atrophy, and macular neovascularisation (MNV) as follows [6]:

- early AMD: medium drusen > 63 μm and ≤125 μm without RPE abnormalities.
- Intermediate AMD: large drusen >125 μm and/or any AMD RPE abnormalities
- Advanced AMD: characterised by the development of either macular neovascularisation (MNV) or geographic atrophy (GA), or both

GA is a distinct clinical finding characterised by the presence of sharply demarcated atrophic lesions of the outer retina caused by loss of photoreceptors, RPE, and the underlying choriocapillaris [7,8]. Typically, these lesions appear initially in the perifoveal region, sparing the fovea first and often increase in size over time until they merge with the fovea [9]. Although this condition is not uncommon in clinical practice, it can go undetected until the atrophy involves the fovea and affects central vision [10,11,12]. Until 2023, there was no proven treatment for GA, placing a significant economic burden on the healthcare system and a major burden on affected patients [13,14]. However, the results of several clinical trials supported the use of anti-complement factor 3 (C3) or anti-complement factor 5 (C5) therapies to reduce GA enlargement [15]. Recently, the results of the OAKS (NCT03525613) and DERBY (NCT03525600) trials in 2022 have confirmed the efficacy of intravitreal pegcetacoplan in slowing the progression of GA lesions [16].

For the treatment of neovascular AMD, intravitreal injections of anti-vascular endothelial growth factor (VEGF) agents are currently used with outstanding anatomic and functional response [17,18,19,20,21]. However, one of the observed features throughout this therapy is the development of RPE and choriocapillaris atrophy, which looks similar to the GA in dry AMD [22].

Many studies have outlined the prevalence of AMD and GA as well as potential risk factors for AMD. However, the results are not entirely consistent [5,23]. Hence, the aim of the present study was to investigate the prevalence of GA in advanced AMD and the proportion of eyes that would meet the indication criteria for treatment with the first upcoming intravitreal anti-C3 agent (pegcetacoplan). In addition, we aimed to investigate factors that might be associated with foveal involvement.

## 2. Methods

This retrospective cross-sectional study assessed a selected group of patients, including all patients: (1) over 50 years of age, (2) diagnosed with any stage of AMD, and (3) who visited the Macular Retina Center MVZ Oberkassel in 2021.

All AMD cases identified were assessed and confirmed by retinal specialists. Following Ferris et al. [6], all eyes were classified into different stages of AMD, involving early, intermediate, and advanced AMD. Eyes with advanced AMD were classified as dry or neovascular AMD (nAMD).

All patients underwent a complete eye examination, including best corrected visual acuity (BCVA), slit lamp biomicroscopy, fundus ophthalmoscopy, infrared reflectance (IR), fundus autofluorescence (FAF), structural en face optical coherence tomography (OCT) and OCT angiography (OCT-A).

Drusen were classified according to their size and degree of borderline sharpness. Hypo- or hyperpigmentation of the RPE was considered an RPE abnormality. FAF was performed in a 30° × 30° visual field centred on the macula.

All eyes underwent en face structural OCT and OCT-A with AngioPlex (Cirrus HD-OCT model 5000; Carl Zeiss Meditec AG, Jena, Germany). A 6 × 6 mm macula-centred scan area was analysed. Each image was created with 350 A-scans in each B-scan and repeated twice in the horizontal and vertical dimensions. All images were acquired using FastTrac retinal tracking technology to minimise motion artefacts. The minimum quality of the OCT-A images was 7 out of 10 [24]. Each participant had then macular cube OCT scan of 128 horizontal B-scans and 512 A-scans per line (6 × 6 mm, fovea-centred). All B-scans were reviewed, particularly to exclude the presence of MNV or retinal fluid.

The atrophic area was identified and measured mainly with en face OCT and then matched with FAF and OCT-A images by a retinal specialist.

**Figure 1:**
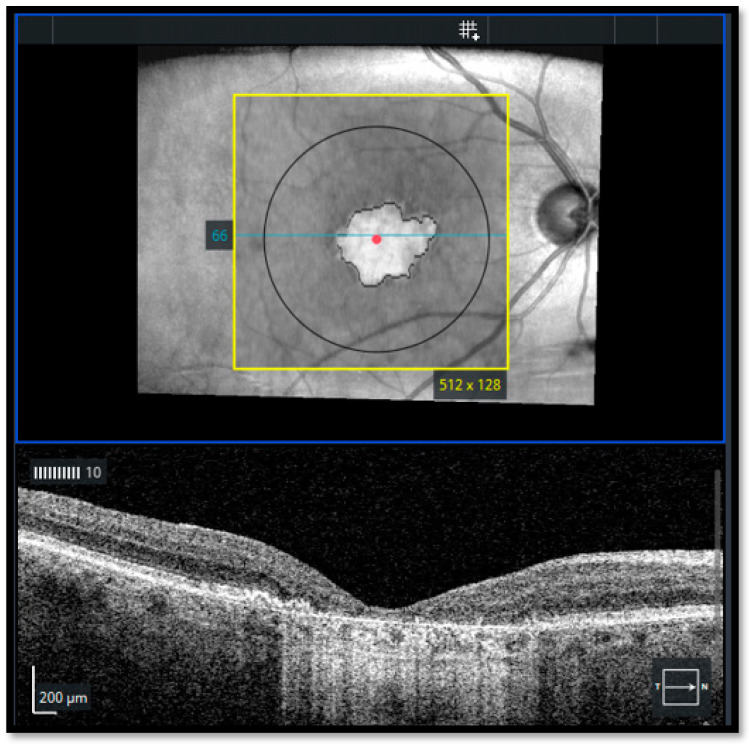
Geographic atrophy as detected by structural en face optical coherence tomography (Cirrus HD-OCT model 5000; Carl Zeiss Meditec AG, Jena, Germany).

- In structural en face OCT, atrophic areas were defined by the advanced RPE analysis programme as sharply demarcated areas of aberrant RPE pigmentation that actually demonstrate a complete atrophy of the RPE and outer retina (cRORA) [25] (Figure 1)
- In FAF, well-defined areas of decreased autofluorescence were manually outlined with the polygon selection tool and considered as atrophic areas and transferred for matching with en face OCT images
- In OCT-A, areas lacking choriocapillaris were classified as atrophic areas using the automatic segmentation of the AngioPlex software with minor manual adjustments

The total GA area was measured by summing all GA areas. To measure GA, the pixel area was converted to square millimetres (mm^2^) based on the image scale. The choriocapillaris under drusen and superficial retinal vessels were excluded to avoid shadowing or residual projection artifacts.

GA could be unifocal or multifocal, but at least one lesion had to have a diameter greater than 188 µm.

Eyes with GA were divided into two groups dependent on the presence or absence of foveal involvement:

- Group 1: eyes with foveal GA (FGA)
- Group 2: eyes with non-foveal GA (NFGA) detected outside the central subfield of the Early Treatment Diabetic Retinopathy Study (EDTRS)

The presence or absence of MNV was determined in all patients by a retinal specialist using a multimodal imaging technique in conjunction with fluorescein angiography for all patients upon initial diagnosis.

MNV was diagnosed in OCT based on the presence of any of the following features: flat irregular RPE elevation (SIRE), subretinal hyperreflective material (SHRM) with poorly defined borders, and intraretinal edema with intraretinal hyperreflective features [26]. The diagnosis was further confirmed on OCTA, the automatic protocol of AngioPlex displays the vascular structure of the choroid, choriocapillaris, deep, and superficial retinal layers. After manual adjustments of segmentation, contrast, and brightness, the presence of MNV complexes was identified as bright, tortuous, or muddled vascular clusters.

Eyes with active MNV were classified as active nAMD, whereas eyes that did not have retinal fluids on OCT at the time of examination but had a history of active MNV (treated with anti-VEGF agents) were classified as non-active nAMD.

MNV activity was assessed by visualising retinal fluid on OCT as a main feature of exudation and angiogenic activity. In addition, and in order to exclude cases of fluid leakage due to other, non-neovascular causes [27], the determination of MNV activity was made after careful assessment depending on:

- Patient history and concomitant diseases
- Previous findings
- Examination of both eyes with multimodality imaging
- Observation of all OCT- B scans
- Evaluation of FAF and OCTA

The main outcome measure was the prevalence of different AMD stages including the prevalence of GA in eyes with advanced AMD.

Based on the OAKS and DERBY studies, the indication criteria for treatment with intravitreal pegcetacoplan were as follows [16]:

- ≥55 years
- GA lesions due to AMD (no other dystrophies)
- BCVA ≥ 1.3 in logarithm of the minimum angle of resolution (logMAR)
- GA lesions with or without subfoveal involvement
- GA size: ≥2.5 and ≤17.5 mm^2^; if multifocal: at least one lesion with a size of ≥1.25 mm^2^
- No MNV in the study eye (active or history of)
- No high myopia (>26 mm/>−6 dpt)
- MNV in the fellow eye was not exclusionary

In addition, some baseline data and clinical factors (age, gender, BCVA, refraction, atrophy area, type of AMD, lens status, and axial length) were compared between eyes with FGA and eyes with NFGA to identify factors that might be associated with foveal involvement.

### 2.1. Ethical Approval

Ethical approval is not required for this type of retrospective, descriptive, non-interventional study. All procedures performed in studies involving human participants were in accordance with the ethical standards of the institutional and/or national research committee and with the 1964 Helsinki declaration and its later amendments or comparable ethical standards. This article does not contain any studies with animals performed by any of the authors.

### 2.2. Statistical Analysis

IBM SPSS version 27 was used for statistical analysis. Continuous data are presented as mean ± standard deviation. Categorical data were summarised as frequencies and percentages and were compared using χ^2^ test. Comparisons between main characteristics were performed by independent Student’s t, Mann–Whitney rank sum, Fisher’s exact, or χ^2^ tests, where appropriate. A *p* value of <0.05 was considered statistically significant.

## 3. Results

### 3.1. Main Characteristics for All Eyes with AMD

**Figure 2:**
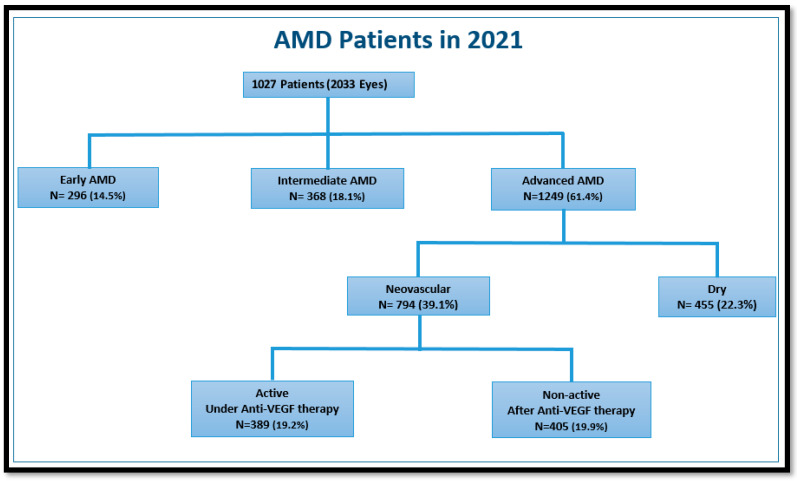
The distribution of all study patients classified according to the different stages of age-related macular degeneration (AMD).

Data were collected from 2033 eyes of 1027 patients. The stage of AMD was early in 296 (14.5%) cases, associated with medium drusen, intermediate in 368 (18.1%) cases, associated with large drusen, advanced in 1249 (61.4%) cases, associated with GA or MNV, and 120 eyes (6%) were found to lack signs of AMD. Of the 1249 eyes with advanced AMD, 455 (22.3%) had dry AMD, while the other 794 (39.1%) AMD eyes had nAMD. Among them, 405 (19.9%) had active nAMD and 389 (19.2%) had non-active nAMD. Quiescent MNV was detected in 11 cases (1.4% of all eyes with nAMD) (Figure 2).

The mean age of all included patients was 76.4 ± 9.5 years, and 62.9% were female. The overall BCVA was 0.49 ± 0.3 in logMAR. The mean spherical equivalent was −0.07 ± 2.0 dpt with a mean axial length of 23.6 ± 1.8 mm. Overall, 68.4% of all eyes examined were pseudophakic. The most frequently documented comorbidities detected during routine ophthalmologic examination were epiretinal membrane (27%) and glaucoma (24%).

The main characteristics of all eyes with AMD, classified according to the different stages of AMD, are listed in Table 1.

### 3.2. Prevalence of GA

There were 1204 (60%) eyes with GA. Among this group, 125 eyes (27.4% of eyes with advanced dry AMD) met the indication criteria for treatment with intravitreal pegcetacoplan based on the OAKS and DERBY studies.

**Figure 3:**
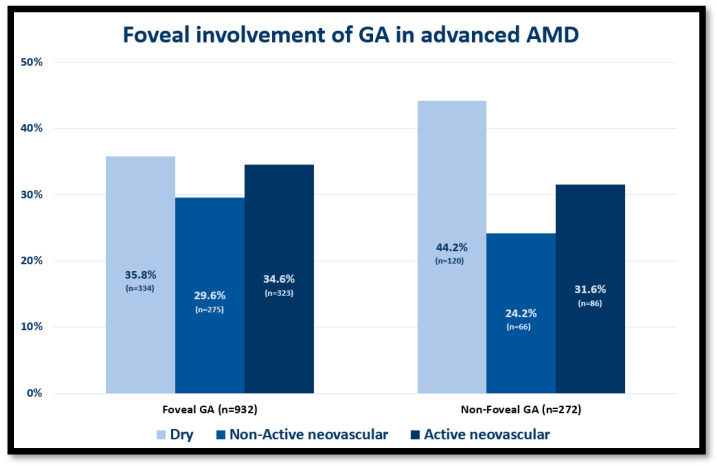
The distribution of eyes with foveal and non-foveal geographic atrophy (GA) classified according to the different types of advanced age-related macular degeneration (AMD).

Of the 1204 eyes with GA, 932 (77%) eyes had FGA, while 272 (23%) had NFGA. There were no significant differences in age, sex, axial length, and lens status between the two study groups. Eyes with FGA had significantly worse BCVA in logMAR [0.8 ± 0.2 vs. 0.4 ± 0.16, *p* = 0.02] and a larger atrophy area [4.2 ± 4.5 vs. 2.2 ± 2.6 mm^2^, *p* = 0.04]. The proportion of eyes with nAMD was significantly higher in the FGA group compared to the NFGA group [598 (64.2%) vs. 152 (55.8%), *p* = 0.01] (Figure 3).

A comparison of the main characteristics between FGA and NFGA in patients with advanced AMD is shown in Table 2.

## 4. Discussion

In the present retrospective cross-sectional study, we investigated the prevalence and stage of AMD including GA in daily practice in a German medical centre. Based on our real-life experience in this study, the most commonly diagnosed stage of AMD was the advanced stage, which can be explained by the fact that patients do not consult an ophthalmologist until they experience visual disturbances.

Although AMD is usually bilateral [28], 10% of patients in our study had unilateral AMD without any AMD findings in the other eye. This percentage might be even higher in Asian patients [29]. In similar studies, the progression of advanced AMD in the fellow eye has been reported to be 20–50% over 5–10 years [30].

In this study, 60% of all AMD eyes had geographic atrophy. The Classification of Atrophy Meetings (CAM) group have provided a new consensus on atrophy where multimodal imaging was combined to define and classify atrophic areas [31]. For this classification, OCT has been proposed as the standard diagnostic method, while other methods, including FAF, IR, and colour imaging provide supporting information. Realising the fact that photoreceptor atrophy can occur without RPE atrophy, four terms have been proposed: complete atrophy of the RPE and outer retina (cRORA), incomplete atrophy of the RPE and outer retina (iRORA), complete atrophy of the outer retina, and incomplete atrophy of the outer retina [31]. In the present study, we defined and measured the atrophic areas with multimodal imaging, mainly en face OCT and then FAF and OCT-A for a more accurate assessment.

On structural en face OCT, the sharply delineated areas of aberrant RPE pigmentation that were detected with the advanced RPE analysis programme effectively demonstrated a complete atrophy of the RPE and outer retina (cRORA).

OCT-A is a new technique that provides the ability to visualise the vascular networks in separate layers including the choriocapillaris plexus. This offers the possibility of detecting GA as loss of choriocapillaris flow beneath atrophic areas [32]. A number of OCTA-based studies have demonstrated impairment of choriocapillaris flow in patients with GA [33,34]. However, the assessment of the choriocapillaris layer can be technically challenging due to artifacts from motion, projections, segmentation errors, and shadow effects caused by large drusen, retinal vessels, and pupil mottling [35,36]. Moreover, choriocapillaris flow deficits are not always synonymous with atrophy, as they may also be dropouts (ischemia) that precede the development of atrophy. Therefore, we used it only as a supplementary tool.

On the other hand, 39.1% of eyes in the present study had nAMD. A total of 51% of them had active nAMD and 49% had non-active nAMD. MNV activity was assessed by visualisation of retinal fluid. Globally, the presence of fluid is still considered to be a universal OCT biomarker for neovascular activity [37]. However, in daily clinical practice, the possibility of confusion, incorrect documentation, and misinterpretation must be anticipated, particularly as some recent studies have provided insight into the various non-neovascular pathways of exudation in eyes with AMD, suggesting that the presence of fluid is not always an indicator of neovascular activity in AMD [38,39,40]. These pathways include cellular degeneration, retinal pigment epithelium dysfunction, pachychoroid disorders, and vitelliform lesions [27].

The distinction between non-neovascular and neovascular fluid is important but not always clear. Degenerative intraretinal cysts can be detected depending on their location on OCT over a region of RPE atrophy and their relative stability over a long follow-up period. Cases of retinal pigment epithelium dysfunction and vitelliform lesions highlight that multimodal imaging, particularly OCTA, is an important tool for distinguishing between non-neovascular and neovascular fluid. For an eye with suspected SRF, the absence of MNV on OCTA can confirm the non-neovascular origin [27].

All of the above indicate a careful assessment of the underlying pathway leading to fluid leakage and involved the examination of both eyes with multimodal imaging, observation of all OCT scans bearing in mind that not all cases of hypofluorescence are associated with fluid leakage, use of different OCT imaging protocols such as volume scan and star scan, and evaluation of FAF and OCTA [41].

In this study, 22.3% of all AMD eyes had geographic atrophy due to advanced dry AMD. A total of 27.4% of these met the clinical trial indication criteria for treatment with intravitreal pegcetacoplan. Pegcetacoplan is a pegylated pentadecapeptide marketed by Apellis Pharmaceuticals for the treatment of complement-mediated disorders. It binds to C3 and its amplification fragment C3b, thereby monitoring the splitting of C3 and the formation of downstream effectors of complement activation [42]. Intravitreal pegcetacoplan showed a significant reduction in the rate of GA area enlargement after monthly or bimonthly treatment compared with randomly allocated controls [16,43]. However, we must be aware that clinical trials often have rigid inclusion criteria that do not always translate to real-world indications, so the estimated prevalence of the potential users of the drug in this study may be underestimated. According to the above, we could reliably predict that at least a quarter of eyes, if not almost all eyes, with GA would be suitable for the upcoming intravitreal pegcetacoplan therapy. This will be administered monthly or every other month in combination with monthly OCT follow-up. Thus, 6–12 injections per eye per year, which rises to the number of applied IVIs in real-life practice. Based on the number of patients in the present study, we expect an additional 1500 IVIs, 1500 postoperative follow-up visits, and 625 OCT examinations per year at our Macular Retina Center.

This huge increase in the number of IVIs and visits requires extraordinary preparation to manage the new expected burden on patients and health care systems. In this context, it is important to bear in mind that these patients have different expectations than patients with nAMD, which will need a special assessment. This demonstrates the epidemiological value of this work, which could be seen as a step in preparation for the upcoming expansion of IVI treatment due to this new treatment for patients with GA.

A further promising treatment option is the use of a novel complement activation site-targeted inhibitor that can be delivered to ocular tissues through subretinal injection of an adeno-associated virus. This results in prolonged release of CR2-fH from the host RPE, which could improve the pathology and visual function [44,45].

Our recent study found that 77% of all eyes with GA had FGA and 23% had NFGA. This discrepancy in prevalence between the study groups could be explained by the fact that patients do not consult an ophthalmologist until they notice visual impairment due to foveal involvement. In addition, this study was conducted in a specialised macular retina centre designed to treat mainly complicated patients with advanced stages of AMD, which implies more cases with foveal involvement.

There were no significant differences in age, sex, axial length, and lens status between these two study groups. However, patients with FGA showed a significantly larger area of atrophy and a worse visual acuity compared to patients with NFGA. This may be due to the role of foveal integrity in determining the visual performance of AMD patients [46]. Preservation of the fovea enables certain visual tasks essential for daily living activities, keeping patients independent for longer and preserving their quality of life [47].

The results of this study also demonstrated that foveal involvement of GA in advanced AMD is more likely in nAMD than in dry AMD. Although GA and MNV are considered different manifestations of advanced AMD, they can coexist in the same eye [48,49,50].

To date, the pathogenesis of atrophy in eyes with nAMD has not been fully elucidated. However, three possible mechanisms may be involved in the development of GA in eyes with MNV, including native progression of the underlying dry AMD, secondary damage due to MNV, and loss of the neurotrophic impact of VEGF secondary to anti-VEGF therapy [51,52,53].

Other clinical studies have reported that the lack of VEGF isoforms has implications for the integrity of the RPE/choriocapillaris complex [54]. Although the relationship between RPE atrophy and choriocapillaris atrophy in AMD is not yet clear, McLeod has shown that in advanced dry AMD, RPE atrophy appears first followed by choriocapillaris atrophy, whereas choriocapillaris atrophy precedes RPE atrophy in nAMD [55]. On the other hand, Grob et al. noted in their study that GA usually occurs before the development of MNV in the combined form [56].

In addition, some clinical studies reported factors associated with an increased risk of developing GA associated with MNV, such as a higher number of injections and the type of MNV and retinal fluids. For example, eyes with intraretinal fluids have an increased risk of developing GA, whereas MNV type 1 has been shown to be more refractory to the progression of GA [57].

Some studies in animal models and postmortem human eyes supported the hypothesis that anti-VEGF agents are significantly associated with the development of GA [57]. However, this association is still controversial in clinical trials. For example, some studies suggest that VEGF inhibition may itself enhance atrophy [58], while others were unable to detect an association between atrophy and treatment frequency [59].

All of this raises the question of whether neovascular and dry AMD should be considered to be one form of advanced AMD rather than two independent clinical forms.

Finally, there are some limitations to this study that need to be taken into account:

- The retrospective cross-sectional design
- Eyes with other ocular comorbidities were not excluded
- GA was not assessed in relation to the number of injections and the type of MNV or retinal fluids

Therefore, given all these issues, further studies with more modern methods are needed to confirm our results.

## 5. Conclusions

This study shows that geographic atrophy in AMD eyes is not uncommon in our daily practice. At least a quarter of eyes with advanced AMD would be suitable for upcoming treatment with intravitreal pegcetacoplan. Patients with foveal geographic atrophy had significantly reduced visual acuity and a larger atrophy area compared to patients without foveal involvement. Foveal involvement of geographic atrophy (GA) in advanced AMD appears to be more likely in neovascular AMD than in dry AMD.

## Figures and Tables

**Table 1:** Main characteristics of all eyes with age-related macular degeneration (AMD), classified according to the different AMD stages.

	All AMD Eyes (N = 1913)	Early (N = 296)	Intermediate (N = 368)	Advanced (N = 1249)
Age (years)	76.4 ± 9.5	73.1 ± 10.1	76.3 ± 8.1	77.6 ± 9.2
Female:Male	62.9%:37.1%	58.8%:41.2%	61.9%:38.1%	62.8%:37.2%
Visual acuity (logMAR)	0.51 ± 0.7	0.15 ± 0.3	0.18 ± 0.2	0.71 ± 0.8
Spherical equivalent (dpt)	−0.07 ± 2.0	−0.41 ± 2.9	0.20 ± 1.6	−0.06 ± 1.7
Cataract:Pseudophakic	28.4%:68.4%	40.2%:54.1%	32.6%:64.6%	24.4%:73.4%
Axial length (mm)	23.6 ± 1.8	24.1 ± 2.1	23.5 ± 1.1	23.5 ± 1.2

**Table 2:** Comparison of main characteristics between eyes with foveal geographic atrophy (GA) and eyes with non-foveal GA in patients with advanced age-related macular degeneration (AMD).

	Foveal (N = 932)	Non Foveal (N = 272)	*p* Value
Age (years)	78.0 ± 9.3	77.8 ± 8.7	0.80
Female: Male	64.3%:35.7%	63.6%:36.4%	0.75
Visual acuity (logMAR)	0.80 ± 0.20	0.40 ± 0.16	0.01
Spherical equivalent (dpt)	0.20 ± 1.7	0.32 ± 1.7	0.80
Atrophy area (mm^2^)	4.2 ± 4.5	2.2 ± 2.6	<0.001
Dry:Neovascular	334:598 (35.8%:64.2%)	120:152 (44.2%:55.8%)	0.01
Cataract:Pseudophakic	214:698 (23%:75%)	76:189 (27.9%:69.4%)	0.08
Axial length (mm)	23.56 ± 1.4	23.59 ± 1.1	0.19

## Data Availability

The datasets used and/or analysed during the current study are available from the corresponding author on reasonable request.
